# Acute effect of meal glycemic index and glycemic load on blood glucose and insulin responses in humans

**DOI:** 10.1186/1475-2891-5-22

**Published:** 2006-09-05

**Authors:** José Galgani, Carolina Aguirre, Erik Díaz

**Affiliations:** 1 Laboratory of Energy Metabolism and Stable Isotopes, Institute of Nutrition and Food Technology (INTA), Universidad de Chile, Chile

## Abstract

**Objective:**

Foods with contrasting glycemic index when incorporated into a meal, are able to differentially modify glycemia and insulinemia. However, little is known about whether this is dependent on the size of the meal. The purposes of this study were: i) to determine if the differential impact on blood glucose and insulin responses induced by contrasting GI foods is similar when provided in meals of different sizes, and; ii) to determine the relationship between the total meal glycemic load and the observed serum glucose and insulin responses.

**Methods:**

Twelve obese women (BMI 33.7 ± 2.4 kg/m^2^) were recruited. Subjects received 4 different meals in random order. Two meals had a low glycemic index (40–43%) and two had a high-glycemic index (86–91%). Both meal types were given as two meal sizes with energy supply corresponding to 23% and 49% of predicted basal metabolic rate. Thus, meals with three different glycemic loads (95, 45–48 and 22 g) were administered. Blood samples were taken before and after each meal to determine glucose, free-fatty acids, insulin and glucagon concentrations over a 5-h period.

**Results:**

An almost 2-fold higher serum glucose and insulin incremental area under the curve (AUC) over 2 h for the high- versus low-glycemic index same sized meals was observed (p < 0.05), however, for the serum glucose response in small meals this was not significant (p = 0.38). Calculated meal glycemic load was associated with 2 and 5 h serum glucose (r = 0.58, p < 0.01) and insulin (r = 0.54, p < 0.01) incremental and total AUC. In fact, when comparing the two meals with similar glycemic load but differing carbohydrate amount and type, very similar serum glucose and insulin responses were found. No differences were observed for serum free-fatty acids and glucagon profile in response to meal glycemic index.

**Conclusion:**

This study showed that foods of contrasting glycemic index induced a proportionally comparable difference in serum insulin response when provided in both small and large meals. The same was true for the serum glucose response but only in large meals. Glycemic load was useful in predicting the acute impact on blood glucose and insulin responses within the context of mixed meals.

## Background

The extent of the postprandial serum glucose response results mainly from the combined effect of the amount and the glycemic index (GI) of carbohydrate contained in a food serving [1,2]. The glycemic load (GL) corresponds to the product of each food item's GI and the amount of carbohydrate in a serving (g) divided by 100. This concept has recently been validated using isolated carbohydrate foods [3,4]. It has been shown that by adjusting the amount of carbohydrate foods in order to obtain identical GL values, a similar blood glucose response is achieved [3]. In addition, stepwise increases in GL produced proportional increases in glycemia [3,4].

When mixed meals containing carbohydrate foods of contrasting glycemic index are consumed, it is known that the difference in postprandial blood glucose response is maintained [5]. However, the magnitude of this differential blood glucose response may be dependent on the meal size.

According to results from studies using isolated carbohydrate foods with contrasting GIs, a higher absolute difference in blood glucose response is anticipated as the meal size increases [6], and, in proportional terms, this difference will be similar at any meal size. This situation can theoretically be predicted by calculating the total GL of a meal. Thus, in meals with equal GI-carbohydrate foods, the absolute difference in blood glucose response will increase as the amount of carbohydrate increases.

We aimed to test these assumptions in the present study by assessing the serum glucose response and other relevant blood variables, after consumption of small and large size meals with contrasting GI. The relationships between the meal GL and serum glucose and insulin responses were also tested. This study showed that meals with two contrasting GIs are equally able to differentially affect the serum insulin responses when provided in a small or large sized meal. Furthermore, direct associations between meal GL and serum glucose and insulin responses were observed.

## Methods

### Subjects

Twelve obese but otherwise healthy women (age 33.2 ± 8.0 (mean and SD) years, weight 82.3 ± 10.6 kg, BMI 33.7 ± 2.4 kg/m^2^) were recruited. Inclusion criteria were absence of clinical signs or symptoms of chronic disease as determined by physical examination and laboratory analyses, not dieting in the preceding 3 months, sedentary life style, not using medication, normal oral glucose tolerance test to rule out diabetes and glucose intolerance [7] and normal fasting lipid profile [8]. All subjects gave their written informed consent to participate in the study. The Institute of Nutrition and Food Technology (INTA) Ethics Board approved the experimental protocol.

### Experimental design

Subjects were asked to avoid any strenuous exercise and maintain their customary dietary intake for 48 h prior to the testing days. On 4 separate occasions, subjects came to INTA on the evening prior to the actual test day. After arrival they ate a standardized dinner containing 34 kJ/kg body mass providing 55% energy as carbohydrates, 25% as fat and 20% as protein. After an overnight fast of 12 h, a blood sampling i.v. cannula was inserted into the antecubital vein. Blood samples were taken at -15, -10 and -5 min (analysed as a pool) before the experimental meal, every 15 min for the first hour and every 30 min thereafter to complete a 5-h postprandial period. All tests were performed within 10 d of the anticipated onset of menses.

### Experimental meals

Meals were served at 08.40 hours and consumed within 20 min. They differed in size (large or small) and type of carbohydrate (high- or low-GI). Thus, the following 4 meals were administered: 1) high-GI/large meal; 2) high-GI/small meal; 3) low-GI/large meal; and 4) low-GI/small meal. Meal size for the large and small meals represented an energy supply equivalent to 49% and 23%, respectively, of the individually predicted basal metabolic rate [9]. In all meals the energy contributed by carbohydrates, fat and protein was 55%, 30%, and 15%, respectively. In order to achieve similar energy density for equal size meals with contrasting GI, water was added to the high-GI meals. Macronutrient composition and foods used in each meal are shown in Table 1. Macronutrient composition was calculated using the Chilean Food Composition Database [10], and the food GI was obtained from published international tables [11]. For each meal, GI and GL were calculated according to the following formulae: GI (%) = ∑(carbohydrate content of each food item (g) × GI)/total amount of carbohydrate in meal (g); GL (g) = ∑(carbohydrate content of each food item (g) × GI)/100. Given the combination of varying total amount of carbohydrate and GI, there were two meals with similar GL (low-GI/large size and high-GI/small size) ultimately resulting in essentially three GL levels (low, medium and high) as shown in Table 1. The assignment of subjects to receive each test meal was randomized first by meal size (small or large) and subsequently by GI (low or high). The first and second test meals in each pair were separated by 2–5 d; the second pair of test meals was given approximately 28 d after the first pair.

**Table 1:** Food composition and nutritional characteristics of the experimental meals.

**High glycemic meals**			**Low glycemic meals**		
	**Large meal**	**Small meal**		**Large meal**	**Small meal**
Liquid 12% fat milk	328 ± 8	153 ± 4	Liquid 12% fat milk	554 ± 13	259 ± 6
White bread	149 ± 3	70 ± 2	Long-grain white rice (boiled 29')	45 ± 1	21 ± 1
Low-fat cheese	37 ± 1	17 ± 0	Low-fat cheese	51 ± 1	24 ± 1
Sucrose	15 ± 0	7 ± 0	Fructose	22 ± 1	10 ± 0
Oil	10 ± 0	5 ± 0	Pear (raw)	133 ± 3	62 ± 1
Butter	3 ± 0	2 ± 0	Bran-cookies	9 ± 0	4 ± 0
Water	287 ± 7	133 ± 3	Oil	14 ± 0	7 ± 0
Energy (kJ)	3208 ± 55	1517 ± 35		3249 ± 74	1516 ± 35
Carbohydrate (g)	111.0 ± 1.9	52.5 ± 1.2		112.3 ± 2.6	52.4 ± 1.2
Fat (g)	22.3 ± 0.4	10.5 ± 0.2		22.6 ± 0.5	10.5 ± 0.2
Protein (g)	30.6 ± 0.5	14.5 ± 0.3		31.0 ± 0.7	14.5 ± 0.3
Dietary fiber (g)	5.4 ± 0.1	2.6 ± 0.1		6.4 ± 0.3	2.9 ± 0.1
Energy density (kJ/g)	4.10 ± 0.00	4.18 ± 0.01		3.92 ± 0.00	3.93 ± 0.02
Glycemic load (g)	95 ± 8	48 ± 4		45 ± 4	22 ± 2
Glycemic index %	85	85		43	43

Mean ± SD

### Blood sample analyses

Venous blood samples for glucose, insulin and FFA were collected in glass tubes and allowed to coagulate on ice for 10 min; serum was then separated at room temperature and stored immediately at -20°C until analysis. Blood glucagon samples were taken in Vacutainer-EDTA with Trasylol^® ^added (50μl/ml of blood), and then plasma was obtained and stored as described above. Serum glucose was assayed by the glucose oxidase method (Photometric Instrument 4010, Roche, Basel, Switzerland). Serum FFA by WAKO NEFA-C test kit (Wako Chemicals, Richmond, VA, USA) on a Hitachi-717 analyser (Tokyo, Japan). Serum insulin was measured using RIA (DSL, Webster, TX, USA). Plasma glucagon was determined by RIA (EURIA-Diagnostica, Malmö, Sweden).

The serum glucose and insulin postprandial responses were assessed using the incremental (iAUC) and total area under the curve (tAUC) at 2 h, 5 h and between 2–5 h. The serum FFA and plasma glucagon postprandial responses were assessed using the tAUC at 2 h, 5 h and between 2–5 h. iAUC and tAUC were geometrically calculated using the trapezoidal method. For the former, area below basal values was not considered [12].

### Statistical analyses

Results are expressed as median and interquartile range, unless stated otherwise. Data showed a non-parametric distribution and were treated as such. The Friedman analysis was used to test between-group differences [13]. In order to determine significance, *post-hoc *testing was performed using the two-tailed Wilcoxon ranked test for paired comparisons [13]. Interactions between type of carbohydrate and macronutrient content were also evaluated. An *alpha *error of 0.05 was considered to be statistically significant. Data were processed with Analyse-It Software, Ltd. (Leeds, UK).

## Results

All subjects completed the experimental protocol; meals were fully eaten in all cases. No complaints or digestive disturbances were observed. Body weight was stable throughout the study (-0.4 ± 1.4%, mean ± SD, p = NS).

### Serum glucose

**Figure 1:**
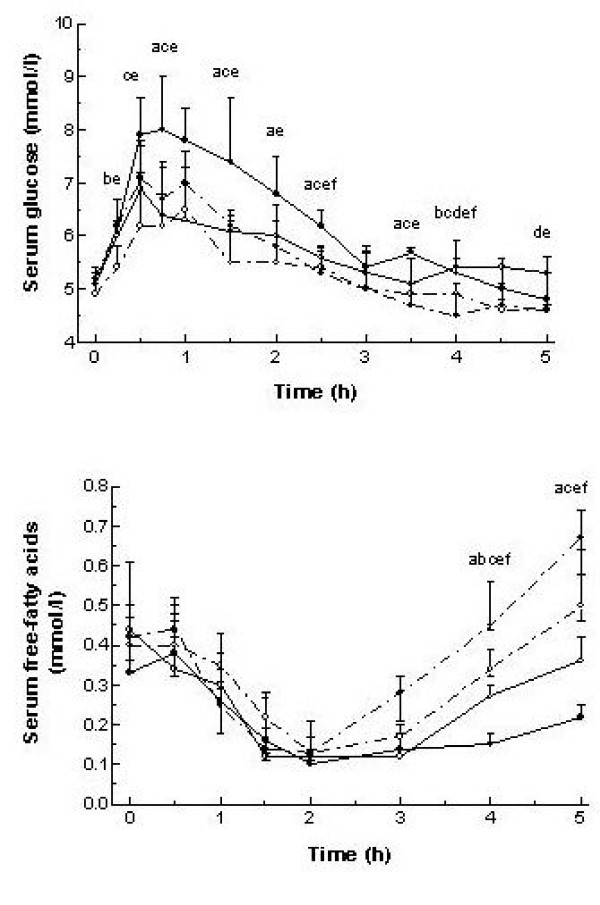
**Serum glucose and free-fatty acid profile aftermeals differing in glycemic index-carbohydrates and meal size**. Values are median and 75^th ^percentile. (∙) high glycemic index; (∘) low glycemic index; (—) large meal size; (----) small meal size. Differences in serum glucose, insulin, FFA and plasma glucagon concentrations were analyzed by Friedman's test and two-tailed Wilcoxon ranked *post hoc *test. Letters indicate significant difference (p < 0.05) between medians as follows: a,  vs ; b,  vs ; c,  vs ; d,  vs ; e,  vs ; f,  vs

The serum postprandial glucose profile showed a similar pattern for all meals, with a peak before 1 h and returning to near fasting values at about 3 h (Figure 1). Meal glycemic index modified serum glucose iAUC and tAUC only with the large meals, whereas no effect was noted after consumption of the small meals. However, when comparing the medians a proportionally similar difference was observed in both cases, particularly when evaluating the serum glucose iAUC. The difference in the serum glucose iAUC was observed at all time periods (0–2 h, 0–5 h, 2–5 h), whereas, for the serum glucose tAUC, the difference was observed in the early postprandial period (0–2 h) only.

**Figure 2:**
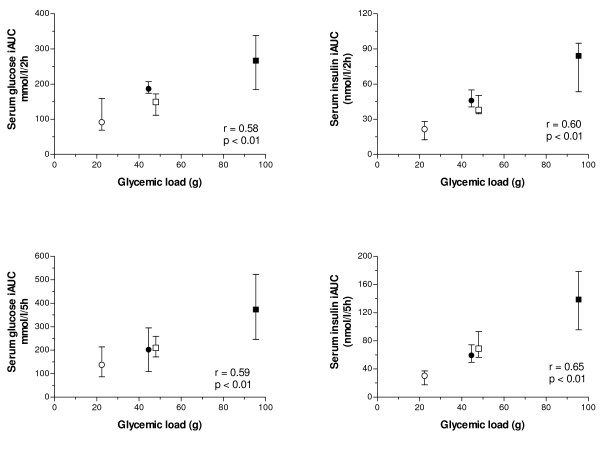
**Relationship between glycemic load and serumglucose and insulin responses over 2 and 5 h**. Values are median and 25^th ^and 75^th ^percentiles. (∘) low-glycemic index/low meal size; (∙) high-glycemic index/low meal size; (□) low-glycemic index/high meal size; (■) high-glycemic index/high meal size.

With regard to the relationship between the estimated GL and observed serum glucose iAUC, a direct association was observed over 2 h (r = 0.58, p < 0.01) and 5 h (r = 0.59, p < 0.01) (Figure 2). A virtually identical association was observed with serum glucose tAUC over 2 h (r = 0.58, p < 0.01) and 5 h (r = 0.58, p < 0.01) (not shown). Likewise, a very similar serum glucose response (as incremental and total AUC) was observed for the low-GI/large meal and high-GI/small meals. This was an expected finding based on the similar GL for these meals (45 and 48 g, respectively).

### Serum free-fatty acids

From fasting to 2 h, postprandial serum FFA suppression was independent of both meal size and carbohydrate GI (Figure 1). Accordingly, no differences were found in serum FFA tAUC over 2 h between meals (p = 0.82, Table 2). From fasting to 5 h, however, serum FFA response differed as a function of the meal size consumed; the response was lower after the large meals compared to the small meals (p < 0.01, Table 2). An influence of GI on serum FFA concentration was observed at 4 and 5 h only (Figure 1). Thus, after the large meals, serum FFA concentration was lower for the high- versus low-GI meal (p < 0.01) while the inverse situation was found after the small meals (p = 0.02).

**Table 2:** Incremental and/or total area under the curve for serum glucose, free-fatty acids, insulin and plasma glucagon after experimental meals.

	**Large meal**		**Small meal**	
	High GI*	**Low GI**	**High GI**	Low GI
tAUC FFA (mmol·l^-1 ^·2 h^-1^)	33 (29–35)	30 (25–35)	34 (31–41)	37 (30–43)
tAUC FFA (mmol·l^-1 ^·5 h^-1^)	63 (56–68)^a^	63 (59–84)^a^	104 (102–109)^b^	91 (80–100)^b^
iAUC Glucose (mmol· l^-1 ^·2 h^-1^)	267 (184–338)^a^	149 (111–172)^b^	187 (74–207)^b^	91 (69–159)^b^
iAUC Glucose (mmol· l^-1 ^·5 h^-1^)	373 (246–523)^a^	215 (176–264)^b^	202 (109–295)^b^	137 (87–214)^b^
tAUC Glucose (mmol· l^-1 ^·2 h^-1^)	866 (771–1000)^a^	743 (720–784)^b^	785 (100–815)^b^	698 (660–745)^b^
tAUC Glucose (mmol· l^-1 ^·5 h^-1^)	1871 (1755–2042)^a^	1705 (1654–1787)^b^	1613 (1577–1770)^b^	1599 (1540–1653)^b^
iAUC Insulin (nmol· l^-1 ^·2 h^-1^)	84 (54–95)^a^	38 (35–50)^b^	46 (40–55)^b^	22 (13–28)^c^
iAUC Insulin (nmol· l^-1 ^·5 h^-1^)	139 (96–178)^a^	69 (57–93)^b^	59 (50–74)^c^	30 (17–37)^d^
tAUC Insulin (nmol· l^-1 ^·2 h^-1^)	97 (67–109)^a^	50 (44–62)^b^	59 (48–69)^b^	34 (30–47)^c^
tAUC Insulin (nmol· l^-1 ^·5 h^-1^)	166 (129–213)^a^	98 (81–136)^b^	87 (79–102)^b^	58 (52–74)^c^
tAUC Glucagon (μg· l^-1 ^·2 h^-1^)	1309 (1100–1424)	1547 (1334–1673)	1452 (1279–1499)	1443 (1189–1686)
tAUC Glucagon (μg· l^-1 ^·5 h^-1^)	3234 (2955–3711)	3677 (3312–4439)	3752 (3141–3885)	3998 (3049–4255)

Median and interquartile range. * Glycemic index. Statistical differences using Friedman's test and two-tailed Wilcoxon ranked *post hoc *test. Medians with different superscript letters in each row are significantly different (p < 0.05).

### Serum insulin

**Figure 3:**
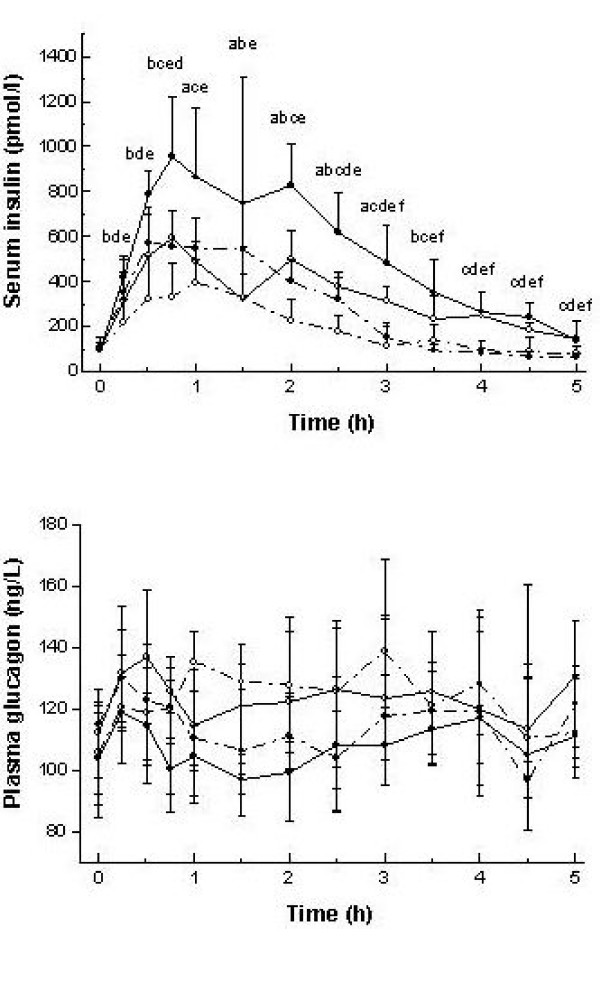
**Serum insulin and plasma glucagon profile after meals differing in glycemic index and meal size**. Values are medianand 75^th ^percentile. (∙) high glycemic index; (∘) low glycemic index; (—) large meal size; (----) small meal size. Differences in serum glucose, insulin, FFA and plasma glucagon concentrations were analyzed by Friedman's test and two-tailed Wilcoxon ranked *post hoc *test. Letters indicate significant difference (p < 0.05) between medians as follows: a,  vs ; b,  vs ; c,  vs ; d,  vs ; e,  vs ; f,  vs

Following the meals, serum insulin profile had a similar shape with a peak before 1 h independent of the GI or meal size, and returning to near fasting levels at about 3 and 5 h for the small and large meals, respectively (Figure 3). The GI influenced the integrated (iAUC and tAUC) postprandial serum insulin responses over 2 and 5 h in both meal sizes (p ≤ 0.016, Table 2). The difference in the serum insulin response for the large meal was observed for all time periods (0–2 h, 0–5 h, 2–5 h) independent of the method of analyzing the postprandial response. On the other hand, for the small meal, the differential effect of GI over 5 h was accounted for in the early postprandial period (0–2 h), when observing both serum insulin iAUC and tAUC. As observed for serum glucose response, a close relationship between GL and serum insulin iAUC was found over 2 h (r = 0.60, p < 0.01) and 5 h (r = 0.65, p < 0.01) with a virtually identical serum insulin response between the two similar GL meals. For the serum insulin tAUC a slightly lower association was observed with the GL over 2 h (r = 0.58, p < 0.01) and 5 h (r = 0.54, p < 0.01) (not shown). In addition, a nearly 2-fold difference in serum insulin iAUC was observed between contrasting GI meals for both meal sizes (Figure 2), whereas for the serum insulin tAUC this difference was between 1.5- to 2-fold.

### Plasma glucagon

Plasma glucagon concentrations were relatively constant during the entire postprandial period (Figure 3). Furthermore, meal size and carbohydrate type did not affect plasma glucagon tAUC over 2 h (p = 0.11) and 5 h (p = 0.10) (Table 2).

## Discussion

This study showed that serum glucose and insulin responses were differentially affected by meals of contrasting GI. However, differences did not reach significance for serum glucose response after the consumption of the small meal. On the other hand, meal GL predicted moderately well the serum glucose and insulin iAUCs and tAUCs. When the amount and type of carbohydrates (together with other macronutrients) present in the meal were adjusted to obtain similar GL values, highly comparable serum glucose and insulin iAUC and tAUC were elicited. In fact, virtually a straight line relationship between the meal GL and serum glucose and insulin responses was found. This relationship was observed despite quite different GI values. With regard to the magnitude of the differences in serum glucose iAUC obtained when meals of contrasting GI are eaten, these can be compared in relative or absolute terms. The relative difference between contrasting GI meals was similar for the small and large meals (about 1.6-fold over 5 h), whilst the absolute difference in median terms was 65 versus 158 mmol· l^-1 ^·5 h^-1 ^for the small and large meals, respectively. These results were predictable from the calculations of GL. This arises from the fact that for foods or meals with equal GI, as the amount of carbohydrate increases, a proportionally constant increase in blood glucose and insulin responses will be observed (e.g. a 2-fold increment in carbohydrate will result in about a 2-fold increase in serum glucose iAUC) [3,4,6]. Whereas when this is compared in absolute terms, the difference is amplified as a function of the increment in the amount of carbohydrate (see formulae in Methods). As a consequence, it is expected that the higher absolute difference is observed for the largest meal (ie, higher GL meal).

When mixed meals are consumed, other food and macronutrients will be present. In this study, the results were similar to those observed in studies using isolated carbohydrates [6] and imply that other macronutrients had a negligible effect on the differential serum glucose and insulin responses. It has, in fact, been reported elsewhere that the amount and type of carbohydrate account for about 90% of the total variability in blood glucose response, whereas protein and fat in mixed meals scarcely contribute to the variance in blood glucose and insulin responses [1,2].

In relation to other blood metabolic responses, this study and others [14-17] demonstrated that mixed meals comprising contrasting GI foods do not, or only slightly affect, the blood FFA response. Only in the late postprandial period (4–5 h) was serum FFA suppression higher for the large versus small meals. This is an expected finding as a function of the higher serum insulin concentration observed during the early postprandial period. In terms of the effect of GI, the result was somewhat unexpected since increased serum FFA concentration was found for the high-GI, small meal. The biological relevance of these findings requires further research as it may be important for understanding disorders of insulin resistance, food intake regulation, and lipid metabolism. With regard to peripheral plasma glucagon levels, as found in other studies, no influence of meal GI or size was observed [14].

An aspect of this study that should be commented is the method (for which many choices exist [18]) chosen to analyze the integrated postprandial response. In order to estimate the food glycemic index in healthy subjects, the Food and Agriculture Organization [19] recommends the use of the incremental AUC, which was corroborated by Wolever [18] after comparing several analysis methods. This recommendation was made based on the fact that the outcome (i.e., GI) was independent of the subjects' characteristics (e.g., diabetic, healthy, etc). On the other hand, when different methods were employed to estimate the change in the blood glucose response before and after a 9-mo exercise program in overweight subjects, Potteiger et al [20] found no differences among the incremental, positive incremental or total AUCs for blood glucose. All of the methods were equally effective in measuring the impact of the intervention on glycemia. In the present study, a virtually identical conclusion was obtained using the incremental and total AUC for both serum glucose and insulin. Critical evaluation of this issue deserves further research.

In conclusion, this study showed that GI alone is unable to predict the glycemic impact when different amounts of carbohydrates are eaten. Furthermore, the use of GL to differentiate the acute impact on blood glucose and insulin responses induced by mixed meals is supported. This is relevant for epidemiological studies investigating the role of carbohydrates in non-communicable chronic diseases.

## Competing interests

The author(s) declare that they have no competing interests.

## Authors' contributions

JG conceived the study, participated in its design and coordination, and helped draft the manuscript. CA performed the study and helped to draft the manuscript. ED conceived the study, participated in its design, and helped to draft the manuscript. All authors read and approved the final manuscript.
